# Experimental and Theoretical Studies on Indigo-Dye-Modified Conjugated Polymers

**DOI:** 10.3390/molecules29133200

**Published:** 2024-07-05

**Authors:** Tionna Douglas, Neetika Singh, Ufana Riaz

**Affiliations:** 1 Department of Chemistry and Biochemistry, North Carolina Central University, Durham, NC 27707, USA; 2 Materials Research Laboratory, Department of Chemistry, Jamia Millia Islamia, New Delhi 110025, India; 3 Department of Materials Engineering, Indian Institute of Science, Bengaluru 560012, India

**Keywords:** indigo, conducting polymer, theoretical studies, UV–visible, fluorescence

## Abstract

The present work reports the synthesis of indigo-dye-incorporated polyaniline (Indigo-PANI), poly(1-naphthylamine) (Indigo-PNA), poly(o-phenylenediamine) (Indigo-POPD), polypyrrole (Indigo-PPy), and polythiophene (Indigo-PTh) via an ultrasound-assisted method. The synthesized oligomers were characterized using FTIR, UV–visible spectroscopy, X-ray diffraction (XRD), scanning electron microscopy (SEM), fluorescence studies, and thermogravimetric analysis (TGA). The experimental data were theoretically compared to analyze the vibrational and electronic spectra via time-dependent density-functional theory (TD-DFT) by applying the Becke, three-parameter, and Lee-Yang-Parr (B3LYP) method with a 6-311G (d,p) basis set. The experimental, theoretical vibrational, and electronic spectra were found to be in close agreement and confirmed the successful incorporation of indigo dye in PANI, PNA, POPD, PPy, and PTh. These studies confirmed that multifunctional oligomers could be synthesized through a facile technique by incorporating dye moieties to enhance their optoelectronic properties, allowing them to be utilized as near-infrared-emitting probes for photodynamic therapy.

## 1. Introduction

Conjugated polymers such as polyaniline (PANI) [1], polycarbazole (PCz) [2], polythiophene (PTh) [3], polypyrrole (PPy) [4], poly(1-naphthylamine) (PNA) [5], poly(o-phenylenediamine) (POPD) [6], etc., have received considerable attention as organic electrochromic materials due to their outstanding photo-physical and optoelectronic properties [7]. Several polymerization techniques, such as copolymerization [8], grafting [9], and functionalization [10,11], have been employed to enhance the extent of conjugation in these polymers, a factor responsible for their remarkable electronic and conductivity characteristics.

Among these methods, dye incorporation is regarded as one of the most facile methods of enhancing the functionality and extent of conjugation for designing polymers with improved optoelectronic properties. The doping of POPD with dyes such as Acid Orange (AO), Fluorescein (Fluo), and Rhodamine-6G (R6G) to design photosensitive and fluorescent polymers has been reported [12]. Similarly, the modification of PANI and POPD with Sudan-I dye has also been carried out via a microwave-assisted method [13]. The quantum yield (*Φ*) values were calculated to be 0.40 and 0.54 for dye-modified PANI and POPD, respectively. Ultrasound-assisted polymerization of azobenzene with aniline, 1-naphthylamine, luminol, and o-phenylenediamine also led to enhanced photo-physical characteristics [14].

Recently, the oligomerization of Bismarck Brown (BB) dye was attempted with luminol, and success was confirmed via experimental as well as theoretical IR and UV-visible studies [15]. The highest occupied molecular orbital (HOMO) and lowest unoccupied molecular orbital (LUMO) energies showed a significant reduction in the band gap upon increasing the content of BB dye.

Indigo and its derivatives have long been used for their intense color and high photochemical stability [16,17]. Electronic materials derived from indolo-naphthyridine have been investigated for their optoelectronics properties, while isoindigo-based conjugated polymers have been designed for the development of semiconducting materials [18,19,20]. Indigo-based polymers containing thermo-cleavable *tert*-butoxycarbonyl (*t*-Boc) groups have shown electron mobility of ~6 × 10^−3^ cm^2^ V^−1^ s^−1^, which is an almost 5-fold increase compared to that of indigo-based polymers alone due to high backbone coplanarity [20]. 

Few studies are available on the modification of polymers/oligomers using dyes to enhance their optoelectronic properties. Moreover, facile techniques for designing polymers/oligomers with controlled emission and enhanced optoelectronic characteristics are highly desirable but have not yet been reported. Hence, with a view to study the effect of indigo dye incorporation on the physico-chemical and optoelectronic properties of synthesized conjugated polymers, the present study reports the synthesis of indigo-dye-incorporated PANI, PNA, PPy, and PTh and POPD via an ultrasound-assisted polymerization technique. The chemical structures of the prepared oligomeric dyes were determined via FTIR studies, and the electronic transitions of polymeric dyes were determined via UV-vis studies and fluorescence measurements. The morphologies of the polymers were confirmed via XRD and SEM analysis. DFT studies were carried out using the DFT/B3LYP method with the 6-311G (d,p) basis set to compare the experimental data.

## 2. Results and Discussion

**Scheme 1:**
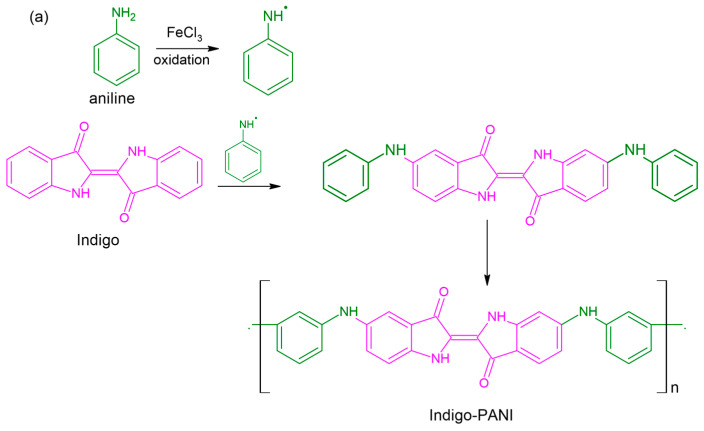

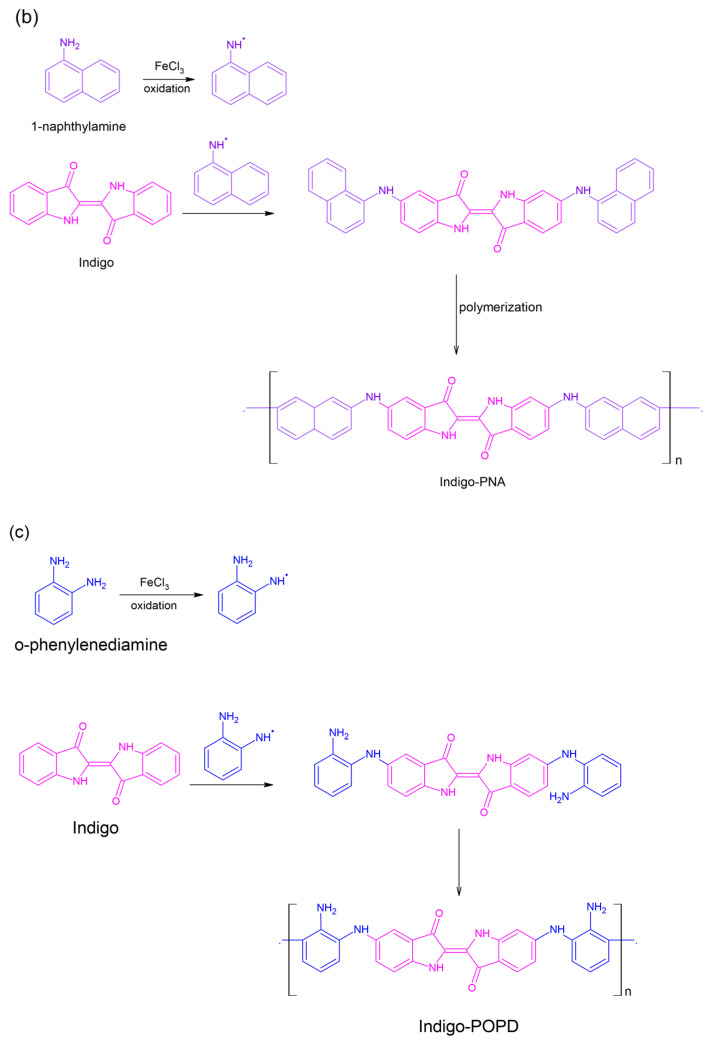

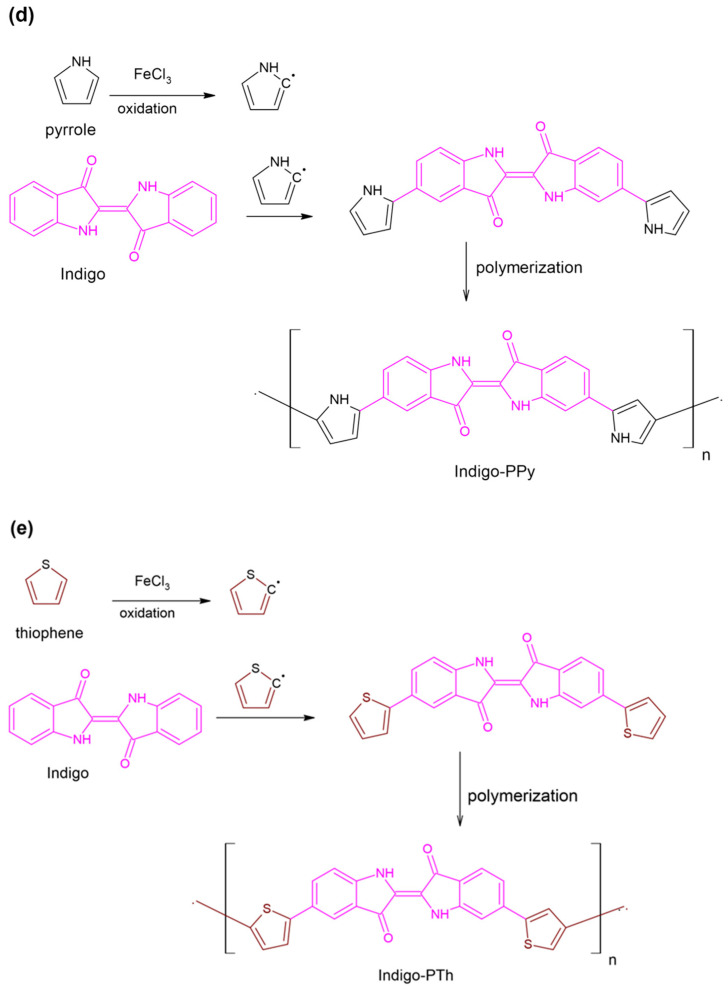
Mechanism of polymerization of (**a**) Indigo-PANI, (**b**) Indigo-PNA, (**c**) Indigo-POPD, (**d**) Indigo-PPy, and (**e**) Indigo-PTh.

The viscosity average molecular weight of the polymers was determined as per the method reported in our earlier studies [10]. The viscosity average molecular weight was determined to be 4568 for Indigo-PANI, 4872 for Indigo-PNA, 5223 for Indigo-POPD, 4876 for Indigo-PPy, and 4321 for Indigo-PTh. The proposed chemical structures are shown in Scheme 1a–e. This scheme reveals that indigo has been incorporated as a dimer due to its greater reactivity with itself than with the monomers. The proposed chemical structures were used for theoretically computing the vibrational and electronic spectra, which were found to be in good agreement with the experimental results. Hence, the proposed mechanism of the insertion of Indigo was acceptable and quite similar to that of other dye-incorporated oligomers shown in our previous studies [13,14,15,16].

### 2.1. Morphological Studies via XRD and SEM Analysis

**Figure 1:**
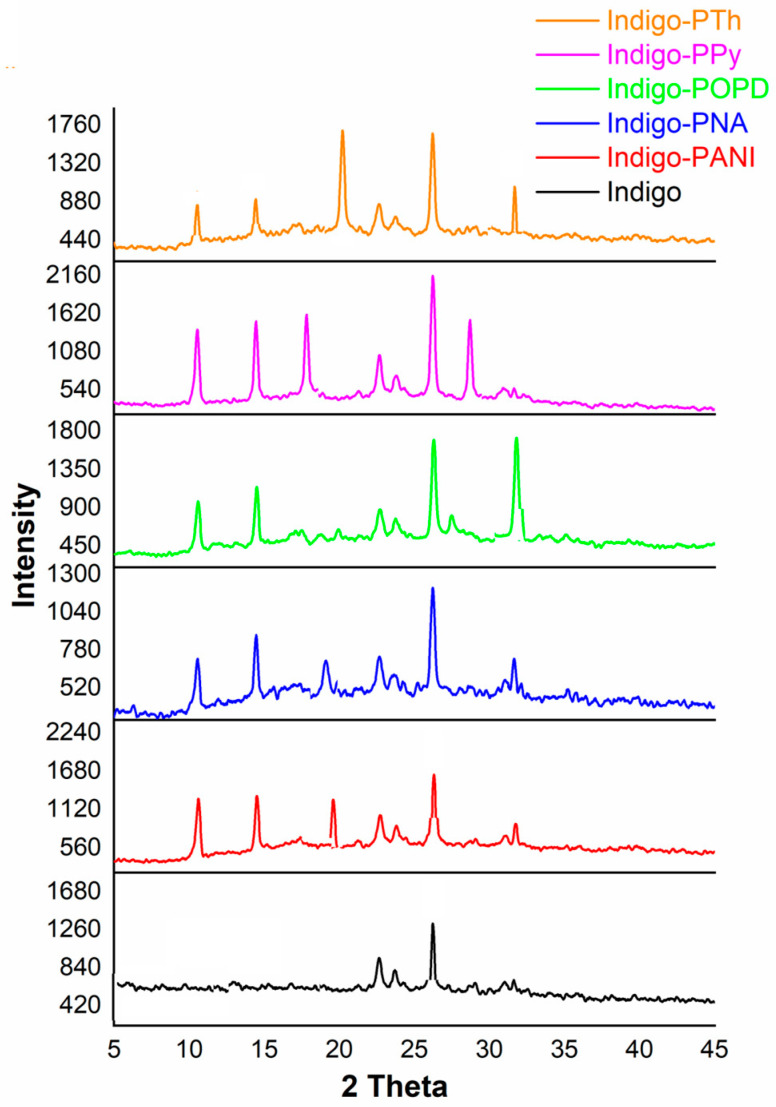
XRD data for Indigo-PANI, Indigo-PNA, Indigo-POPD, Indigo-PPy, and Indigo-PTh.

XRD was used to explore the crystallinity of synthesized oligomers. The XRD profile of Indigo dye, shown in Figure 1, revealed peaks at 2*θ* = 22.71°, 23.93° and 26.34°. The XRD profile of Indigo-PANI revealed additional peaks at 2*θ* = 10.61°, 14.59°, 19.26°, and 31.79°, besides the peaks corresponding to the presence of Indigo dye at 2*θ* = 22.71°, 23.93°, and 26.34°. The new peaks were correlated with the planes associated with PANI, while the peaks corresponding to Indigo dye revealed the same intensity as that in the pristine XRD profile of the dye, which confirmed that no major structural reorganization occurred upon the incorporation of Indigo dye in PANI. The XRD profile of Indigo-PNA displayed peaks at 2*θ* = 10.76°, 14.54°, 18.23°, 22.71°, 23.76°, 26.50°, and 31.63°. The polymer revealed semi-crystallinity like that noticed in Indigo-PANI. The XRD profile of Indigo-POPD exhibited peaks ranging from 2*θ* = 10.47 to 31.93°, while Indigo-PPy revealed peaks ranging from 2*θ* = 10.76° to 31.17°. The XRD profile of Indigo-PTh also revealed peaks in the same range. The incorporation of Indigo in PANI, PNA, POPD, PPy, and PTh led to a minor shifting of peaks, and the appearance of new sharp peaks confirmed the presence of well-formed planes in the polymers [13]. The incorporation of Indigo contributed to crystallinity and did not result in any structural reorganization for any of the dye-modified polymers [12,13].

**Figure 2:**
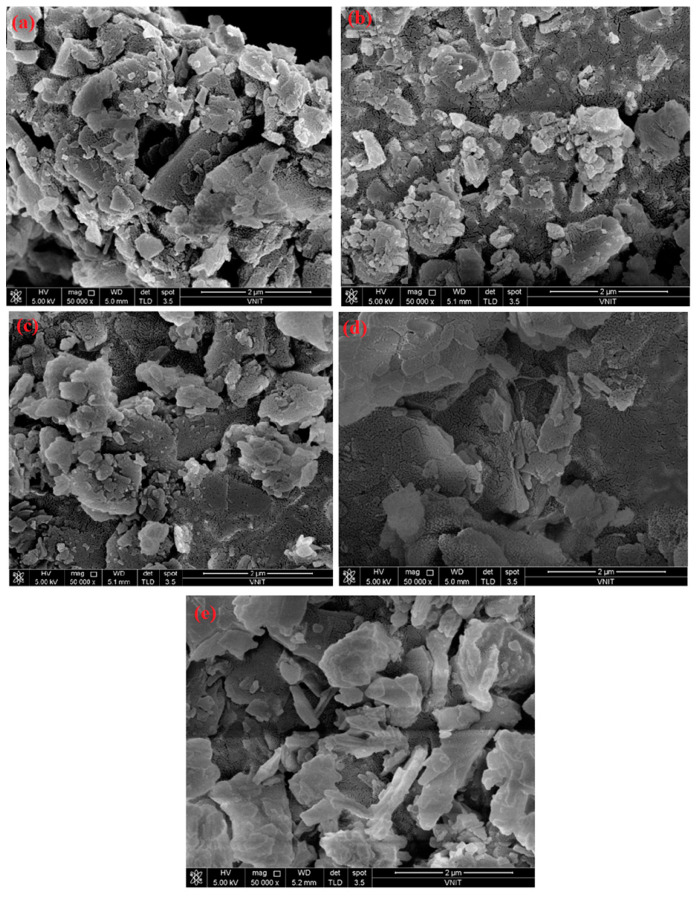
SEM images of (**a**) Indigo-PANI, (**b**) Indigo-PNA, (**c**) Indigo-POPD, (**d**) Indigo-PPy, and (**e**) Indigo-PTh.

The SEM data for Indigo-PANI (Figure 2a) show the formation of a two-dimensional (2D) jagged sheet-like structure, while the SEM data for Indigo-PNA, shown in Figure 2b, reveal a granular aggregated morphology of variable sizes. The SEM results for Indigo-POPD, shown in Figure 2c, and Indigo-PPy, shown in Figure 2d, exhibit a flaky structure with highly stacked clusters. The SEM results for Indigo-PTh, shown in Figure 2e, reveal the formation of elongated rods mixed with flaky agglomerates. The morphology appeared to be crystalline in all cases, and this fact was also corroborated by the XRD results. The morphology was found to vary for all the oligomers, confirming that the type of monomer into which Indigo was incorporated governed the morphology in all cases. Indigo-PPy showed different huge flaky structures. Indigo-PTh showed distorted, rod-like structures embedded within it along with a granular morphology. Hence, it can be concluded that the selection of the appropriate monomer could be used to control the architecture of the oligomers.

### 2.2. Thermogravimetric Analysis

**Figure 3:**
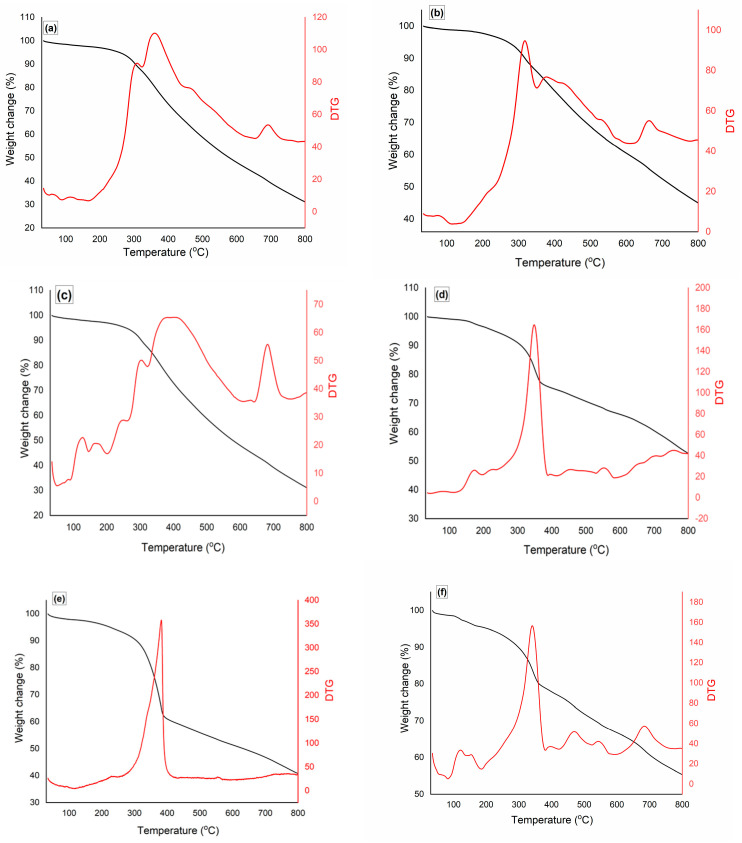
TGA-DTG profiles of (**a**) pure indigo dye, (**b**) Indigo-PANI, (**c**) Indigo-PNA, (**d**) Indigo-POPD, (**e**) Indigo-PPy, and (**f**) Indigo-PTh.

The TGA profiles of Indigo dye, Indigo-PANI, Indigo-PNA, Indigo-POPD, Indigo-PPy, and Indigo-PTh are depicted in Figure 3a–e. The TGA profile of pure indigo dye, shown in Figure 3a, revealed 5 wt.% loss around 100 ℃ and 20 wt.% loss at 400 ℃ corresponding to the decomposition of the carbonyl group.

The DTG profiles showed a broad exotherm around 100 °C and a sharp endotherm at 400 °C, results correlated with the decomposition of the dye molecule through the fragmentation of carbonyl groups. Around 35 wt.% loss was noticed at 500 °C, while 70 wt.% decomposition was noticed at 800 °C. The TGA profile of Indigo-PANI, shown in Figure 3b, also revealed almost 2 wt.% loss around 100 °C due to evaporation of moisture, whereas 10 wt.% loss was noticed around 298 °C, and the DTG showed a sharp exothermic event, which was correlated with structural reorganization of the polymer prior to decomposition. Almost 30 wt.% loss was achieved at 500 °C, and 55 wt.% loss was observed at 800 °C, confirming the polymer to be thermally stable. Likewise, The TGA profile of Indigo-PNA (Figure 3c) showed the occurrence of multiple exothermic events prior to 400 °C, and a broad exotherm was noticed around this temperature, showing almost 20 wt.% degradation. The presence of multiple exotherms spanning between 150 °C and 350 °C could be correlated with structural reorganization of the oligomeric chains prior to decomposition, and almost 68 wt.% loss was noticed at 800 °C. The polymer was found to be less stable than Indigo-PANI. The TGA profile of Indigo-POPD (Figure 3d) showed 25 wt. % loss at 350 °C and a sharp exothermic event around this temperature. The maximum loss of 45 wt.% was noticed at 800 °C, and the polymer showed higher thermal stability than Indigo-PNA and Indigo-PANI. Interestingly, the TGA profiles of Indigo-PPy, shown in Figure 3e, exhibit a sharp and pronounced exothermic event at 375 °C corresponding to 40 wt.% loss of the polymer due to the decomposition of the dye moiety as well as the heterocyclic rings, while 60 wt.% decomposition took place at 800 °C. The TGA profile of Indigo-PTh, shown in Figure 3f, reveals multiple exothermic events spanning from 100 to 200 °C, 200 to 300 °C, 400 to 500 °C, and 600 to 700 °C, confirming that the polymer had a highly branched structure, which presumably caused rapid structural reorganization at every decomposition temperature. Almost 20 wt.% decomposition was seen at 350 °C, while 45 wt.% decomposition took place at 800 °C. The TGA-DTG profiles clearly revealed that the polymers were highly stable.

### 2.3. Fluorescence Studies

**Figure 4:**
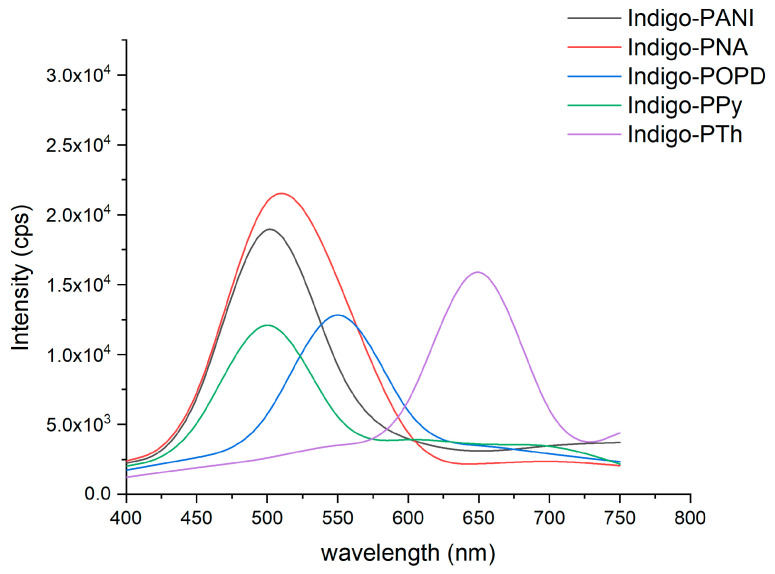
Fluorescence emission spectra of Indigo-PANI, Indigo-PNA, Indigo-POPD, Indigo-PPy, and Indigo-PTh.

The fluorescence emission spectra of the Indigo-dye-modified polymers (excited at 380 nm) are depicted in Figure 4. The emission spectrum of Indigo-PANI revealed an intense peak at 500 nm, while the emission spectra of Indigo-PNA also exhibited intense emission at 510 nm upon excitation at 380 nm. The emission spectrum of Indigo-POPD showed a broad peak at 550 nm, and the emission spectra of Indigo-PPy and Indigo-PTh revealed peaks at 480 nm and 600 nm, respectively. The quantum yields (*Φ*) of the oligomers were calculated as per the reported method, using Rhodamine B as a reference [15,16,17,18]. The *Φ* values obtained were 3.2 × 10^−4^, 6.2 × 10^−3^, 5.3 × 10^−3^, 4.0 × 10^−4^, and 1.6 × 10^−3^ for Indigo-PANI, Indigo-PNA, Indigo-POPD, Indigo-PPy, and Indigo-PTh, respectively. The highest quantum yield was observed for Indigo-PNA, while the lowest quantum yield was noticed in the case of Indigo-PPy, as shown in Table 1. This could be attributed to the structural orientation and twisting of the oligomeric chains upon the insertion of Indigo dye, which lowered the quantum yield values.

### 2.4. Comaprison of Experimental Data with Computational Studies to Confirm the Proposed Structures of Indigo-Dye-Modified Oligomers Using DFT: Optimized Geometries and Frontier Molecular Orbitals

**Figure 5:**
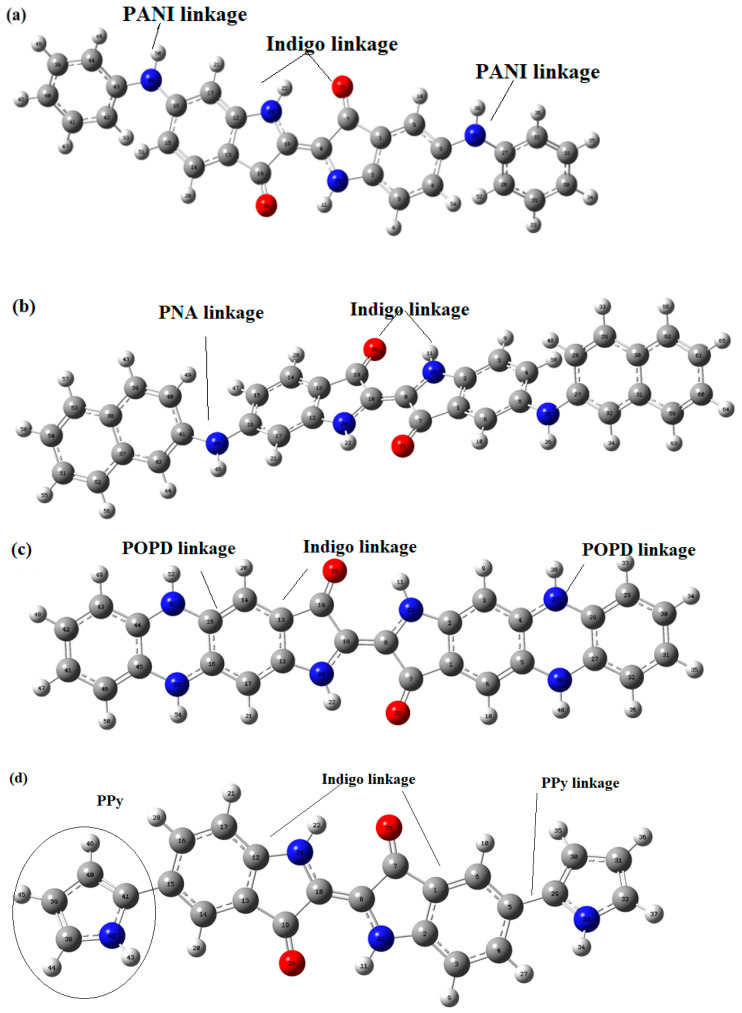

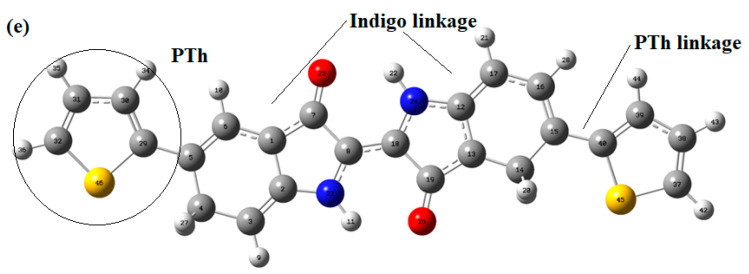
Optimized geometries of (**a**) Indigo-PANI, (**b**) PNA, (**c**) Indigo-POPD, (**d**) Indigo-PPy, and (**e**) Indigo-PTh, Sulfur (yellow), Carbon (grey), oxygen (red), Nitrogen (blue), Hydrogen (white).

The Indigo-dye-modified oligomers were optimized by inserting one monomeric unit of dye and two units of monomer flanked on either side of the dye molecule, as shown in Figure 5a–e. The optimized geometry of Indigo-PANI, depicted in Figure 5a, showed a planar configuration. The optimized geometry of Indigo-PNA, shown in Figure 5b, exhibited a twisted configuration. The optimized geometries of Indigo-POPD, Indigo-PPy, and Indigo-PTh, shown in Figure 5c–e, also revealed a planar configuration with no visible twisting upon insertion of the Indigo dimer.

The Muliken charge distribution for Indigo-PANI, Indigo-PNA, and Indigo-POPD (given in Figure S1a–c in the Supporting Information) was found to be centered around the N atoms of the amide NH- group as well as on the C=O groups of the Indigo dye. For Indigo-PPy, shown in Figure S1d, the charge was found to be concentrated around the NH of PPy and the C=O groups of the Indigo dye, while for Indigo-PTh, shown in Figure S1e, it was noticed to be centered around the C=O groups of Indigo and also around the C–C bonds linked to the S group.

**Figure 6:**
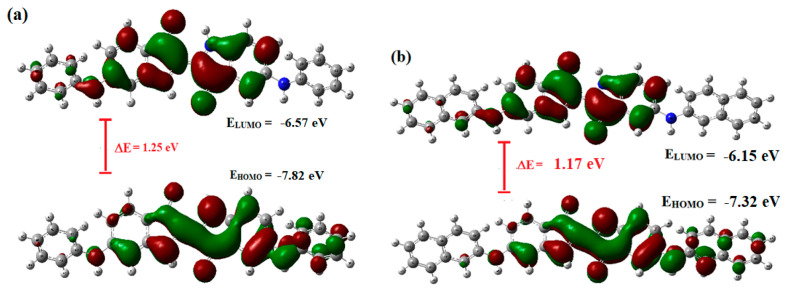

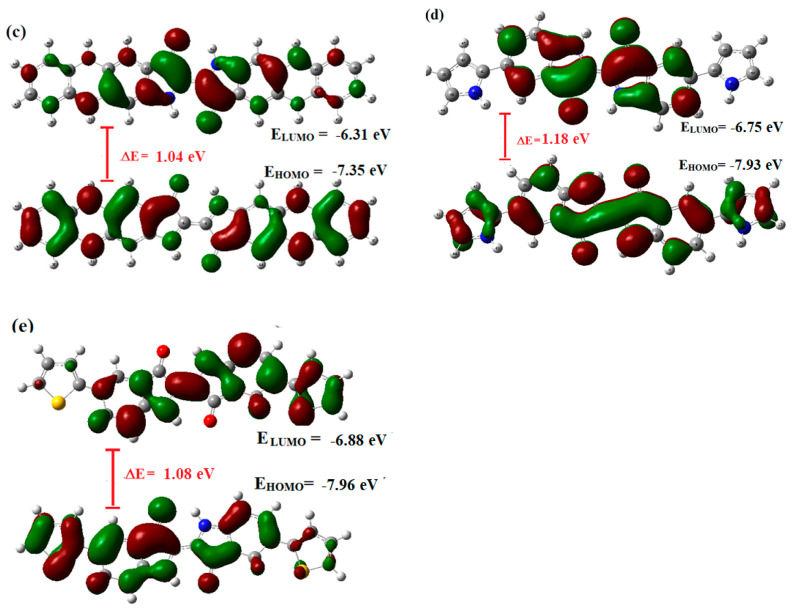
HOMO-LUMO energies of (**a**) Indigo-PANI, (**b**) Indigo-PNA, (**c**) Indigo-POPD, (**d**) Indigo-PPy, and (**e**) Indigo-PTh.

The frontier molecular orbitals were computed to explore the changes in the bandgap upon insertion of Indigo dimer in the oligomers. The highest occupied molecular orbital (HOMO) of Indigo-PANI, shown in Figure 6a, was found to be evenly distributed over the backbone of the dye moiety, while the lowest unoccupied molecular orbital (LUMO) appeared to be less symmetric and completely distributed along the aniline ring. The HOMO-LUMO energy was computed to be 1.25 eV. For Indigo-PNA, shown in Figure 6b, the LUMO orbitals were noticed to be concentrated around C–C linkage of Indigo dye, and the 1-napththylamine monomer ring and the HOMO orbitals were uniformly distributed over the Indigo dimer. The HOMO-LUMO energy was determined to be 1.17 eV and lower than that of Indigo-PANI due to the presence of a fused benzene ring, which increased the extent of conjugation in the oligomer. The oligomer Indigo-POPD, depicted in Figure 6c, showed symmetrically distributed HOMO/LUMO orbitals, and the energy was computed to be 1.04 eV. Likewise, for Indigo-PPy and Indigo-PTh, shown in Figure 6d,e, the HOMO-LUMO distribution was quite symmetrical and concentrated around the linkage connecting the dye and the monomer molecule. The HOMO-LUMO energies were computed to be 1.18 eV and 1.08 eV for Indigo-PPy and Indigo-PTh, respectively. The incorporation of Indigo was found to result in variable HOMO-LUMO energy values, which were found to be significantly lower than the band gap values of the pristine polymers reported in the literature [14]. A similar observation of lowering of energy gap values was also made in our earlier studies pertaining to dye incorporation in polymers [14,15]. The dye moieties have conjugated bonds in their structures, and their incorporation into the oligomers/polymers enhances conjugation, which lowers the energy gap values.

### 2.5. Comparison of Expermental and Theoretical UV–Visible Spectra

**Figure 7:**
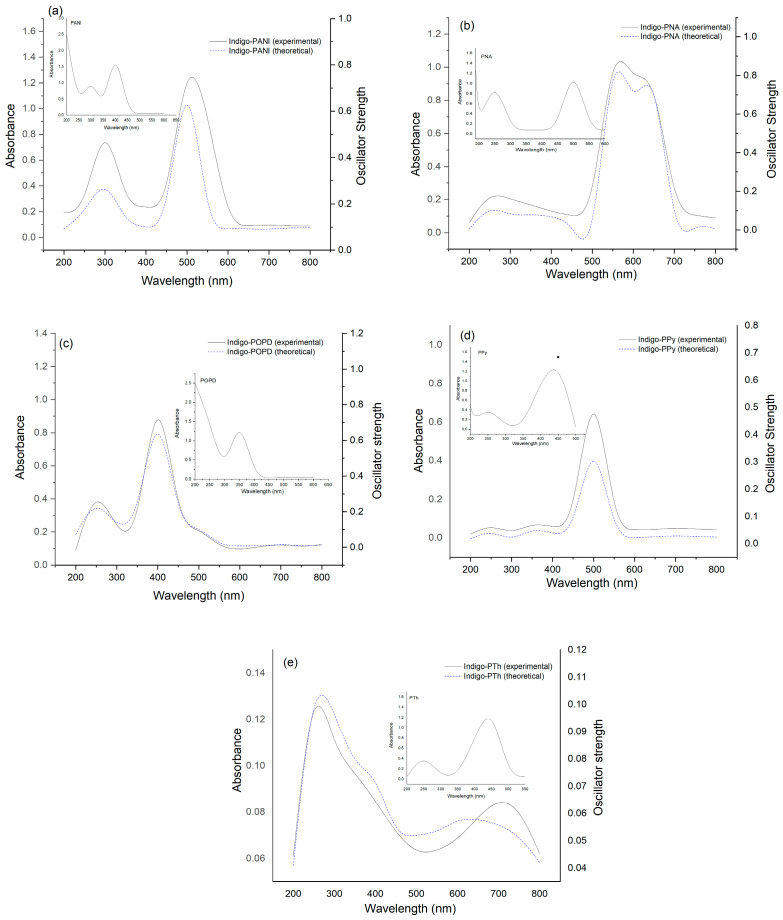
UV-Vis spectra of (**a**) Indigo-PANI, (**b**) Indigo-PNA, (**c**) Indigo-POPD, (**d**) Indigo-PPy, and (**e**) Indigo-PTh. (inset in Figures refers to UV spectra of pristine olymers in absence of Indigo dye).

The changes in the electronic transition in the oligomers upon incorporation of Indigo dye were studied experimentally as well as theoretically, as shown in Figure 7. The experimental UV–visible spectrum of pure Indigo dye revealed peaks at 250 nm, 350 nm, and 600 nm (given in Figure S2 in the Supporting Information). The peaks in the UV range are due to π-π*, while those in the visible range are due to n-π* transition. The spectrum of PANI in the absence of Indigo (given in the inset) showed peaks at 275 nm and 400 nm, while the experimental UV spectrum of Indigo-PANI, shown in Figure 7a, exhibited peaks at 280 nm in the UV range due to π-π* and at 500 nm in the visible range due to the n-π* transition, the latter of which is also referred to as polaronic band [14]. Polarons/bipolarons are charge carrier defect states formed by p/n-type doping through the π-conjugated backbone. The π-polaron band arises due to π-delocalization present in the conducting polymer. The degree of delocalization will be greater if the conjugation increases and hence result in the shifting of π-polaron band towards higher wavelengths [21]. The theoretical spectrum also revealed peaks in the same range showing an oscillator strength value of 0.33 for the polaronic peak. The shifting of the polaronic peak was due to the greater extent of conjugation induced by the insertion of Indigo dye. The UV spectrum of Indigo-PNA, shown in Figure 7b, displayed peaks at 285 nm, and a broad hump spanning between 500 and 700 nm and the theoretical spectrum of Indigo-PNA revealed the n-π* transition peak at 500 nm, with an oscillator strength value of 0.70. The spectrum of PNA in the absence of Indigo revealed peaks at 250 nm and 500 nm. Likewise, The UV spectrum of POPD in the absence of Indigo showed a pronounced peak at 350 nm, whereas the experimental UV spectrum of Indigo-POPD, shown in Figure 7c, revealed peaks at 250 nm and 400 nm. The theoretical spectrum of the same oligomer revealed a pronounced peak at 400 nm with an oscillator strength value of 1.4 [10]. This intense peak corresponded to the n-π* transition. The UV spectra of Indigo-PPy and Indigo-PTh (shown in Figure 7d,e) displayed peaks at 250 nm, 500 nm, and 700 nm, corresponding to π -π* and n-π* transitions, respectively. The theoretical spectra showed n-π* transitions matching the experimental spectra. The oscillator strength for the 500 nm peak in Indigo-PPy was computed to be 0.45, while the oscillator strength for the 700 nm peak in Indigo-PTh was computed to be 0.25. The oligomers showed variation in the electronic transitions upon modification with Indigo dye, which suggests that desirable electronic transitions could be achieved by varying the monomer and incorporating Indigo dimer [13,14]. The experimental and theoretical data were found to be in close agreement with each other for all the dye-modified oligomers.

### 2.6. Comparison of Experimental and Computational Vibrational Spectra

The IR spectral data of Indigo-PANI (given in Table S1 and Figure S3a–e in the Supplementary Information) showed characteristic peaks at 3258 cm^−1^ and 3032 cm^−1^ corresponding to the N–H stretching vibration. The peak at 1620 cm^−1^ was ascribed to carbonyl stretching (C=O), while the peak at 1613 cm^−1^ was attributed to C=C conjugated alkene stretching [13,14,15]. The C=C quinonoid stretching peaks were observed at 1585 cm^−1^, 1483 cm^−1^, and 1456 cm^−1^, and the C=C benzenoid stretching peaks were seen at 1390 cm^−1^, 1336 cm^−1^, 1313 cm^−1^, and 1298 cm^−1^. Multiple peaks at 1256 cm^−1^ and 1219 cm^−1^ were correlated with the C–N stretching vibration mode. The characteristic peaks at 1198 cm^−1^, 1170 cm^−1^, 1124 cm^−1^, 1104 cm^−1^, 1092 cm^−1^, 1065 cm^−1^, and 1010 cm^−1^ were correlated with the C–H bending mode. Aromatic ring stretching vibrations were noticed between 934 cm^−1^ and 656 cm^−1^. The IR spectral data of Indigo-PNA revealed the N–H stretching peak at 3249 cm^−1^ and 3056 cm^−1^. The peak at 1623 cm^−1^ was ascribed to carbonyl stretching (C=O), and the peak at 1610 cm^−1^ was correlated with the C=C conjugated alkene stretching [14,15]. The C=C quinonoid stretching peaks appeared at 1586 cm^−1^, 1482 cm^−1^, and 1460 cm^−1^, while the C=C benzenoid peaks were found at 1391 cm^−1^, 1334 cm^−1^, 1315 cm^−1^, and 1296 cm^−1^. The C–N stretching peak was noticed at 1222 cm^−1^, while the presence of multiple peaks between 1186 cm^−1^ and 1006 cm^−1^ were correlated with C–H bending. Aromatic ring stretching vibrations were noticed between 946 cm^−1^ and 662 cm^−1^ [13,14,15]. The IR spectral data of Indigo-POPD showed N–H stretching peaks at 3258 and 3032 cm^−1^ and the carbonyl stretching (C=O) peak at 1622 cm^−1^. C=C quinonoid stretching peaks were noticed at 1586 cm^−1^, 1482 cm^−1^, and 1457 cm^−1^, while C=C benzenoid peaks showed up at 1389 cm^−1^, 1337 cm^−1^, 1317 cm^−1^, and 1297cm^−1^. C–N stretching peaks appeared at 1249 cm^−1^ and 1221 cm^−1^. C–H bending peaks were found between 1198 cm^−1^ and 1009 cm^−1^_,_ while aromatic ring stretching vibrations were noticed between 940 cm^−1^ and 664 cm^−1^. The IR spectral data of Indigo-PPy showed the N–H stretching characteristic peak at 3268 cm^−1^, while the C=O stretching peak appeared at 1625 cm^−1^. C=C stretching peaks were seen between 1585 and 1298 cm^−1^. C–N stretching peaks were found at 1254 cm^−1^ and 1220 cm^−1^. Similar peaks were noticed in the case of Indigo-PTh. The presence of multiple NH stretching peaks, C=O stretching, CN vibration peaks, and quinonoid/benzenoid peaks confirmed the polymerization and successful modification of the polymer with Indigo dye [13,14,15]. It was interesting to note that the theoretical data closely matched the experimental values, which confirmed the proposed structures of the polymers, as depicted in Scheme 1a–e.

## 3. Conclusions

Indigo-dimer-incorporated oligomers of PANI, PNA, POPD, PPy, and PTh were successfully synthesized via an ultrasound-assisted technique. The XRD results confirmed the semi-crystalline characteristics of the oligomers, and SEM revealed a remarkable transformation of the morphology from a flaky to mixed morphology of granules and rod-like structures upon incorporating Indigo dye. Fluorescence emission was observed to be in the near-infrared region for Indigo-PTh and around the visible region for the other oligomers. The theoretical results obtained using DFT studies were observed to be in close agreement with the experimental IR as well as UV data. The frontier molecular orbital distribution showed a reduction in HOMO-LUMO energy upon the incorporation of Indigo dye. This technique is a facile way of introducing multifunctionality in oligomers/polymers via the incorporation of dyes that usually have a conjugated architecture. This method could be applied in designing near-infrared-light-emitting probes utilized in photodynamic therapy.

## 4. Experimental Section

Indigo (GLR Innovations, New Delhi, India), chloroform (Merck, New Delhi, India), ferric chloride (Merck, New Delhi, India), thiophene (Loba Chemie Pvt. Ltd., New Delhi, India), pyrrole (Merck, New Delhi, India), Aniline (Sigma Aldrich, St. Louis, MI, USA), ortho-phenylenediamine (Sigma Aldrich, St. Louis, MI, USA), 1-naphthylamine (Loba Chemie Pvt. Ltd., Maharashtra, India), dimethyl sulfoxide (DMSO) (Merck, New Delhi, India), dimethylformamide (DMF) (S.d. Fine Chem., Pvt. Ltd., New Delhi, India), *N*-Methyl-2-pyrrolidone (NMP) (Merck, New Delhi, India), and distilled water were used without further purification.

### 4.1. Synthesis of Indigo-Dye-Incorporated Polymers

Aniline (40 mL, 4.3 × 10^−1^ mol) dissolved in chloroform (50 mL) was added to a round-bottom flask (250 mL) containing Indigo dye (5 g, 1.9 × 10^−2^ mol) and sonicated in an ultrasonic bath (model Scope Enterprises Pvt. Ltd., India) between 0 °C and 5 °C. Ferric chloride (3.1 g, 1.9 × 10^−2^ mol) was added as an initiator to the reaction mixture and further sonicated for 5 h at the same temperature. The obtained polymer was then kept in a deep freezer for 24 h at −5 °C and subsequently centrifuged, washed several times with distilled water using an R-8C laboratory centrifuge, and dried in a vacuum oven for 24 h at 70 °C. The same reaction was carried out using 1-naphthylamine, o-phenylenediamine, pyrrole, and thiophene, and the synthesized polymers were designated as poly Indigo-PANI, Indigo-PNA, Indigo-POPD, Indigo-PPy, and Indigo-PTh, as shown in Scheme 1a–e. (% yield: Indigo-PANI: 88%, Indigo-PNA: 82%, Indigo-POPD: 78%, Indigo-PPy: 76%, and Indigo-PTh: 80%; solubility: partially soluble in ethanol, methanol, chloroform, and H_2_O and completely soluble in dimethylsulphoxide (DMSO), N-methyl pyrrolidone (NMP), and dimethyl formamide (DMF).)

### 4.2. Characterization

#### 4.2.1. Spectral Analysis

IR spectra of conjugated polymers were obtained using an FT-IR spectrophotometer (Shimadzu, Model IRA Affinity-1, Kyoto, Japan), while ultraviolet–visible light (UV-vis) spectra were recorded using a UV-vis spectrophotometer (Shimadzu UV-1800, Kyoto, Japan), using a water/NMP mixture as a solvent. Fluorescence studies were performed using a fluorescence spectrophotometer (Fluorolog @ 3-11, Bengaluru, India), using water/NMP as a solvent.

#### 4.2.2. Morphological Analysis

XRD patterns of the conjugated polymers were recorded on Malvern Pananalytical, (USA Philips model PW 3710 2400 Computer Drive, Suite 2100, Westborough, MA, USA) using Ni-filtered Cu-Kα radiation. TEM micrographs were obtained using a Morgagni model 268-D TEM system (Hillsboro, OR, USA).

#### 4.2.3. Thermal Analysis

TGA analyses of the polymers were carried out using STA 6000 (Perkin Elmer Instruments, Shelton, CT, USA) at a heating rate of 10 ℃/min over a temperature range of 50–800 ℃ in a nitrogen atmosphere.

#### 4.2.4. DFT and TD-DFT Calculations

The calculations were performed using GAUSSIAN 09 software(Wallingford, USA) and the optimized geometries were obtained using DFT/B3LYP method with the 6-311G (d,p) basis set [12,13]. The oscillator strength, HOMO-LUMO energies, and band gap were determined using the optimized geometries with the same basis set. The same optimized structures were used in the vibrational frequency calculations. The UV−vis spectra of the geometry-optimized structures were simulated at TD-DFT/B3LYP using 6-311G (d,p) basis set.

## Figures and Tables

**Table 1:** Fluorescence quantum yields of Indigo-PANI, Indigo-PNA, Indigo-POPD, Indigo-PPy, and Indigo-PTh.

Polymers	Peak Position (nm)	Area	Quantum Yield
Indigo-PANI	500	8.8 × 10^4^	3.2 × 10^−4^
Indigo-PNA	510	2.2 × 10^6^	6.2 × 10^−3^
Indigo-POPD	550	4.6 × 10^6^	5.3 × 10^−3^
Indigo-PPy	480	1.8 × 10^5^	4.0 × 10^−4^
Indigo-PTh	650	8.0 × 10^4^	1.6 × 10^−3^

## Data Availability

Data is included in the manuscript and supporting information.

## Supplementary Materials

The following are available online at https://www.mdpi.com/article/10.3390/molecules29133200/s1. Figure S1: Muliken charge distribution in (a) Indigo-PANI, (b) Indigo-PNA, (c) Indigo-POPD, (d) Indigo-PPy, (e) Indigo-PTh, Figure S2: UV visible spectrum of pure Indigo dye, Figure S3: experimental and theoretical IR spectra of (a) Indigo-PANI, (b) Indigo-PNA, (c) Indigo-OPD, (d) Indigo-PPy, (e) Indigo-PTh, Table S1: IR spectra data of Indigo-PANI, Indigo-PNA, Indigo-POPD, Indigo-PPy and Indigo-PTh.
