# Supplementary material for: Experimental and Theoretical Studies on Indigo-Dye-Modified Conjugated Polymers
Source: Molecules. 2024 Jul 5;29(13):3200. doi: 10.3390/molecules29133200 (PMC11243738; doi:10.3390/molecules29133200)
Supplement: Supplementary file 1 [file molecules-29-03200-s001.zip › molecules-3048345-supplementary.pdf]

Supporting Information

Experimental and Theoretical Studies of Indigo Dye Modified Conjugated Polymers

Tionna Douglas [a] , Neetika Singh [b,c] and Ufana Riaz [a,b*]

[a] Department of Chemistry and Biochemistry, North Carolina Central University, NC, 27707, USA, *Corresponding author email: ufana2002@yahoo.co.in
[b]Materials Research Laboratory Department of Chemistry, Jamia Millia Islamia, New Delhi-110025, India,
[c]Department of Materials Engineering, Indian Institute of Science, Bengaluru-560012, India

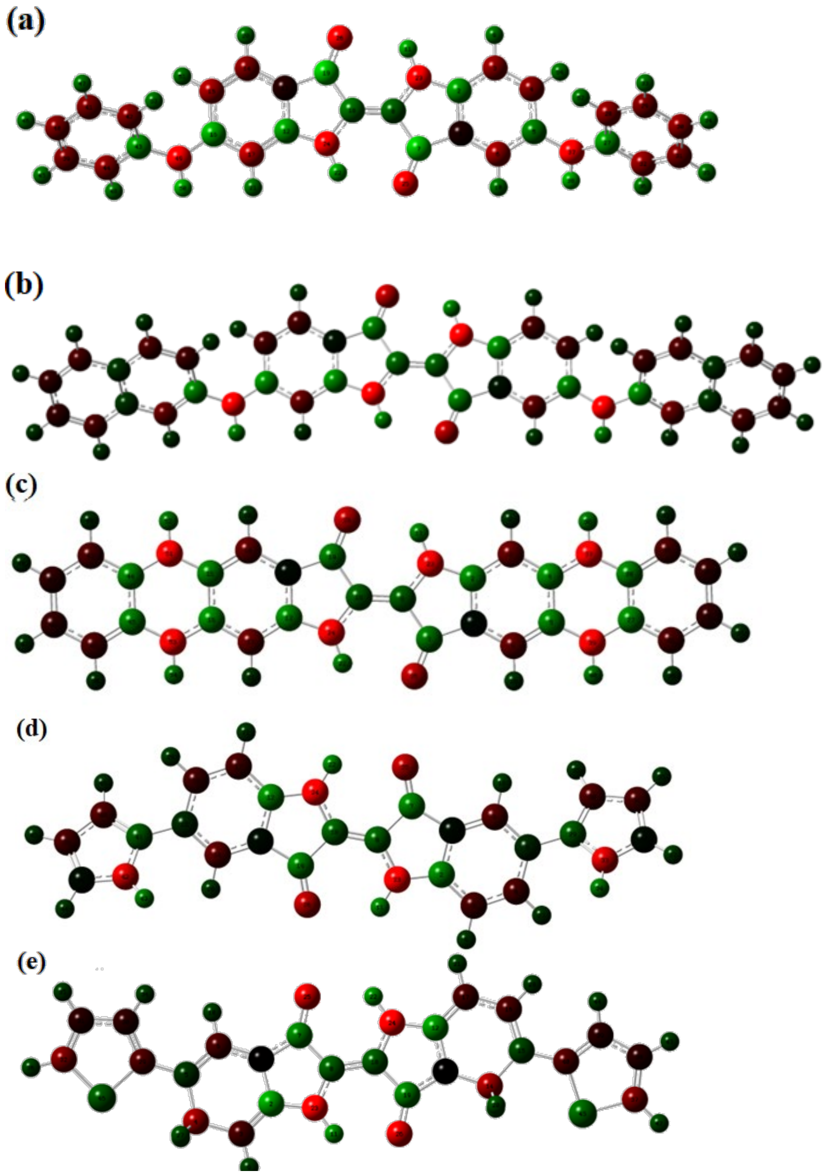

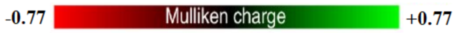

Figure S1. Muliken charge distribution in (a) Indigo-PANI, (b) Indigo-PNA, (c) Indigo-POPD, (d) Indigo-PPy,(e) Indigo-PTh

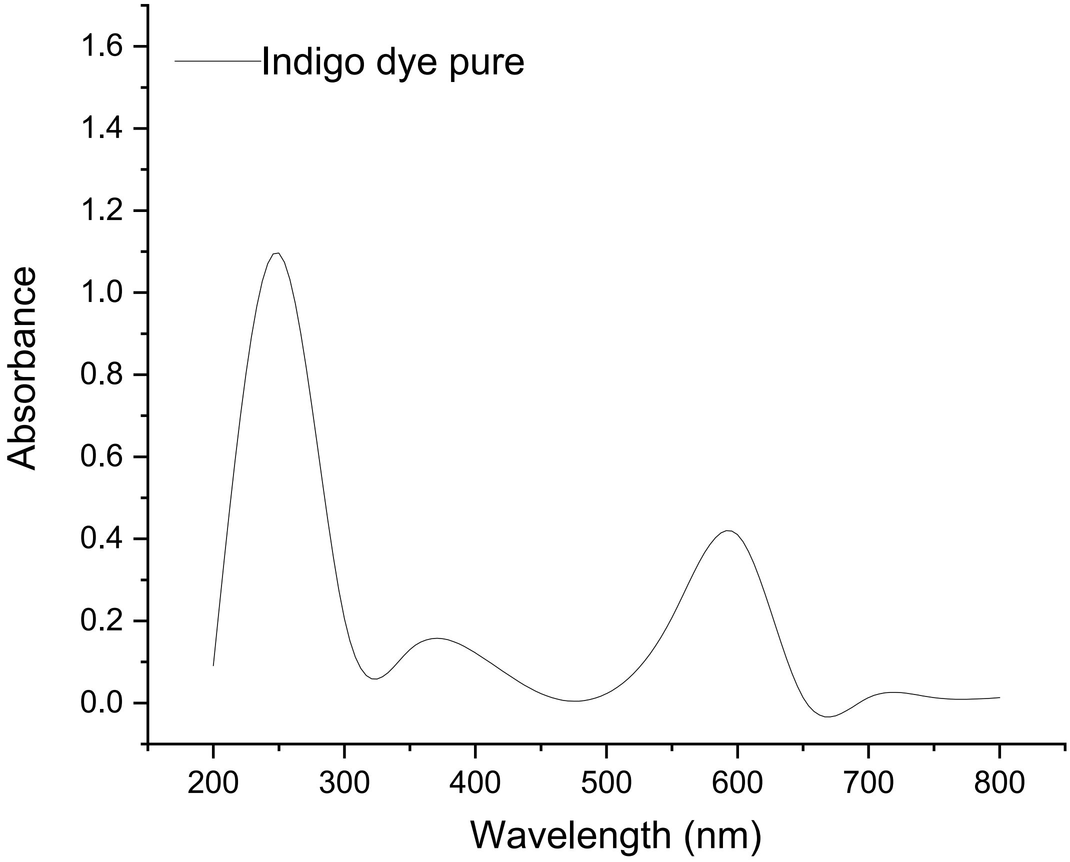

Figure S2. UV visible spectrum of pure Indigo dye

(a)

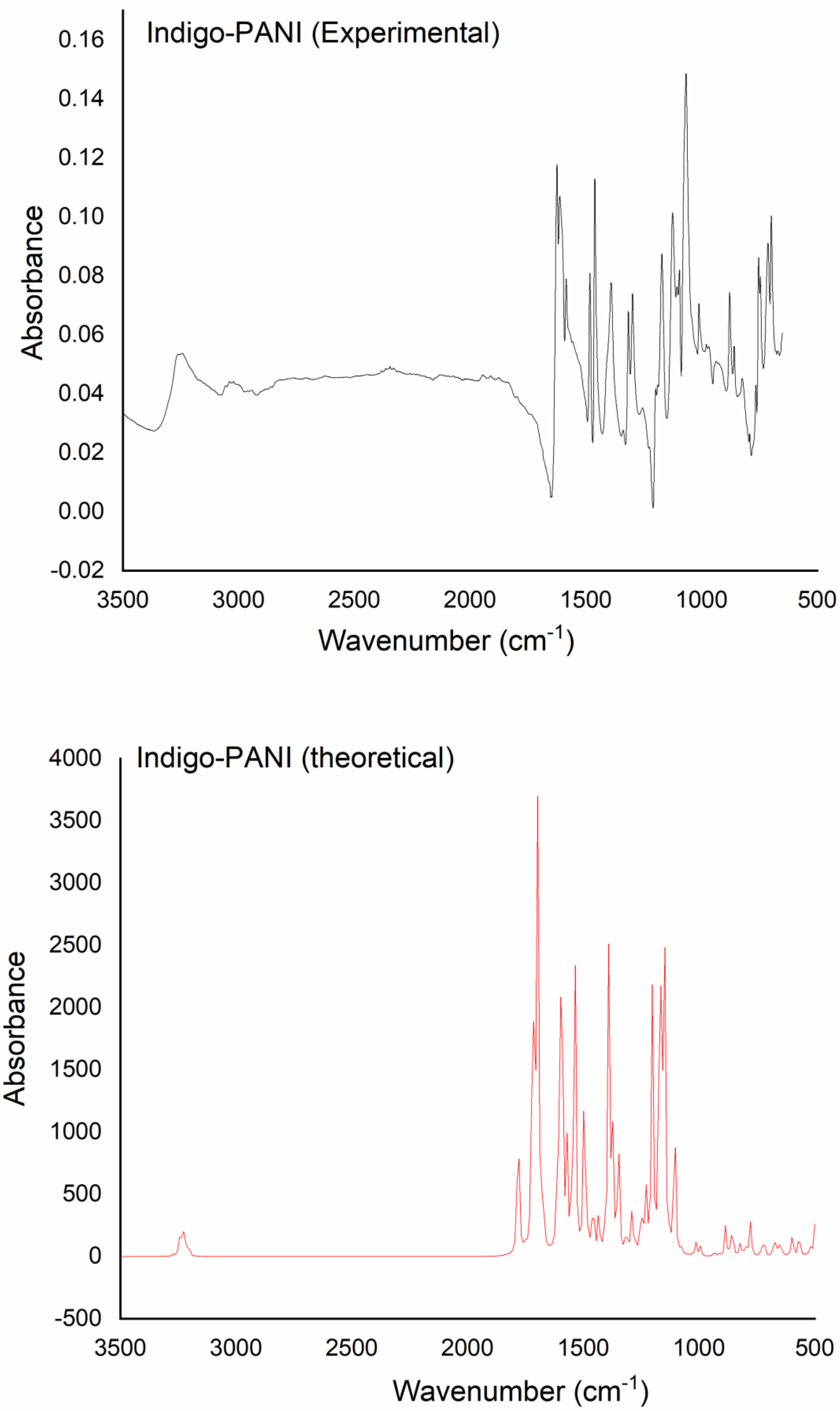
Indigo-PANI (Experimental)
Absorbance
0.16
0.14
0.12
0.10
0.08
0.06
0.04
0.02
0.00
-0.02
3500
3000
2500
2000
1500
1000
500
Wavenumber (cm-1)
Indigo-PANI (theoretical)
Absorbance
4000
3500
3000
2500
2000
1500
1000
500
0
-500
3500
3000
2500
2000
1500
1000
500
Wavenumber (cm-1)

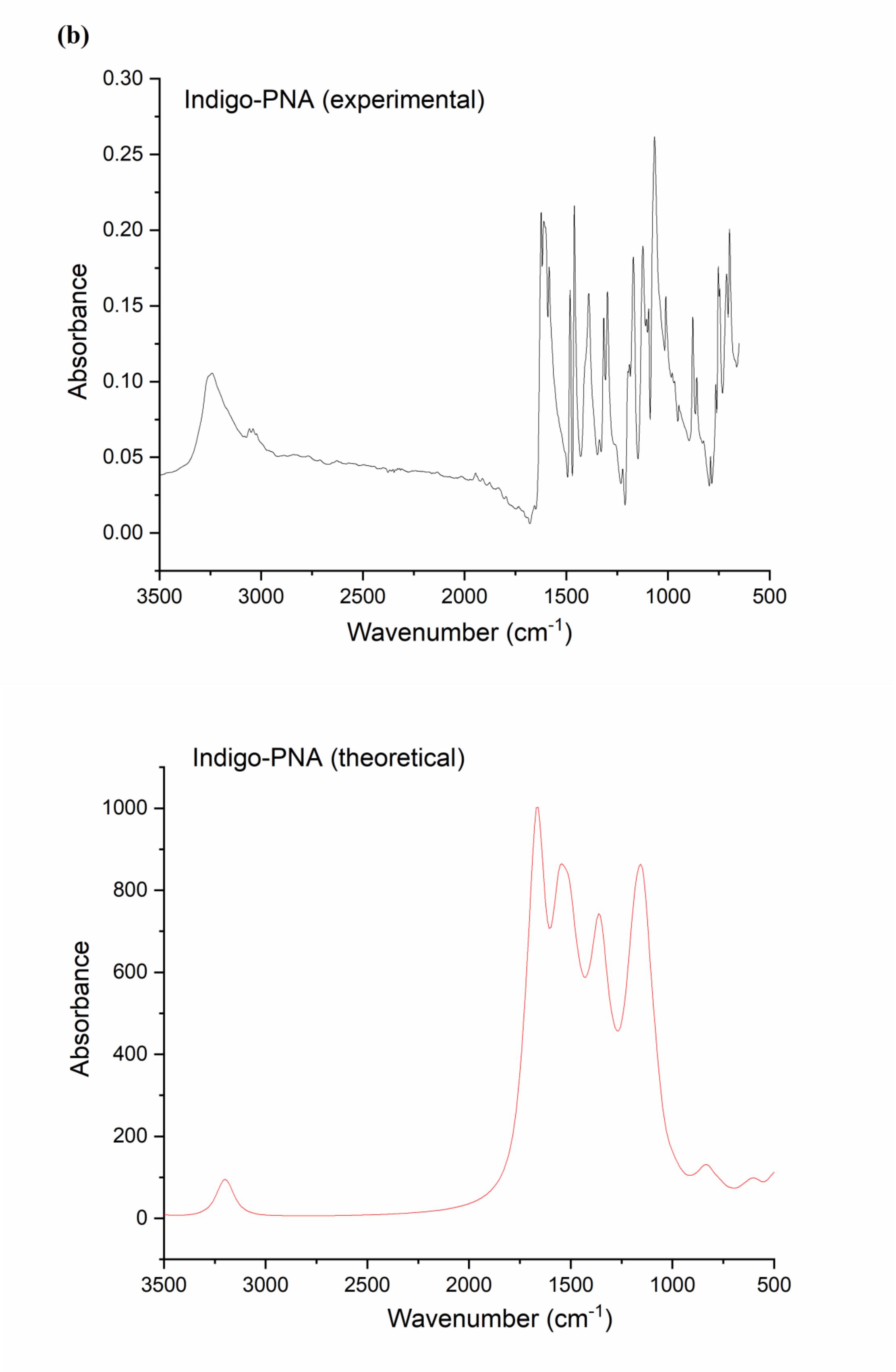
(b)
Indigo-PNA (experimental)
Absorbance
0.30
0.25
0.20
0.15
0.10
0.05
0.00
3500
3000
2500
2000
1500
1000
500
Wavenumber (cm-1)
Indigo-PNA (theoretical)
Absorbance
1000
800
600
400
200
0
3500
3000
2500
2000
1500
1000
500
Wavenumber (cm-1)

(c)

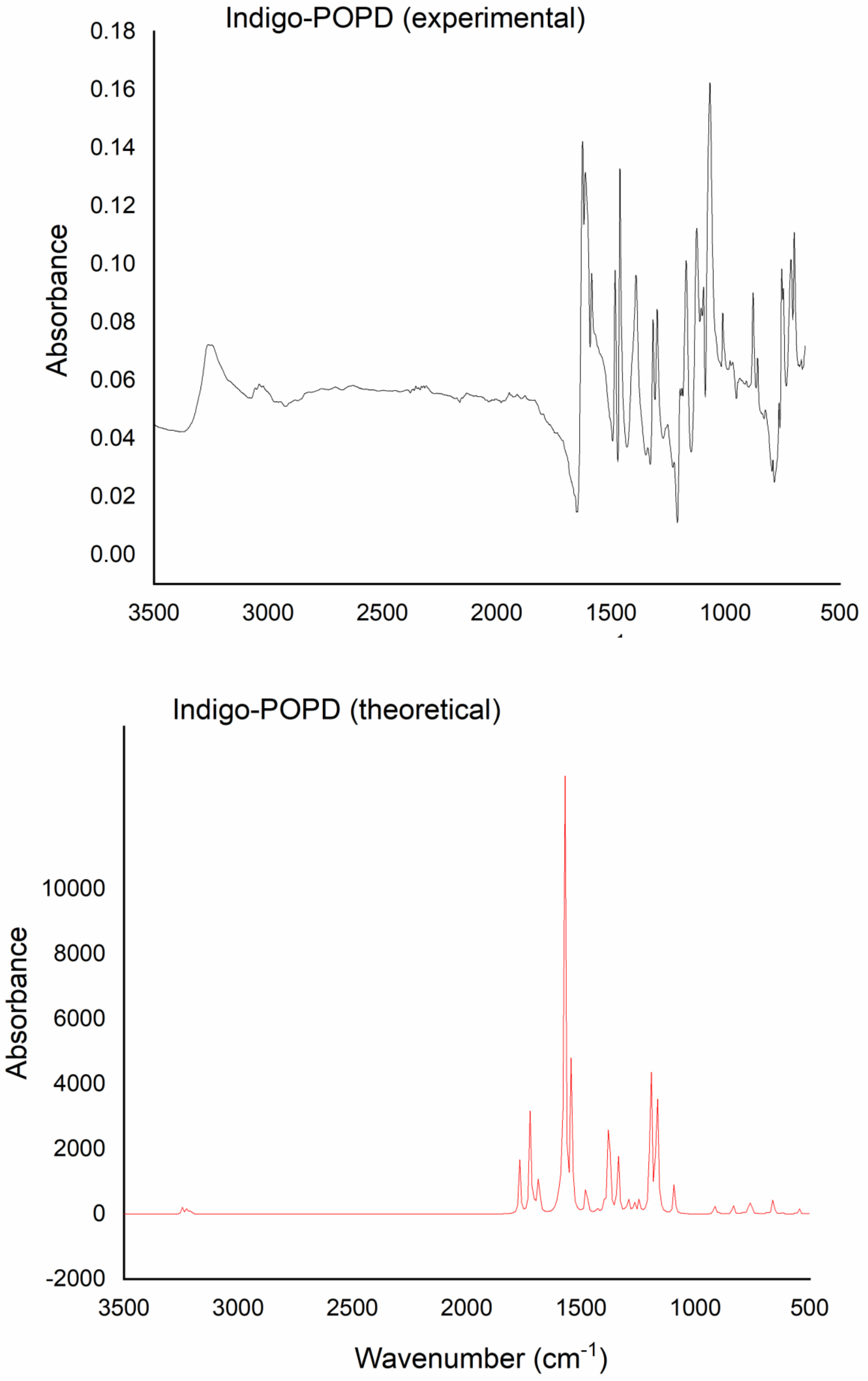
Indigo-POPD (experimental)
Absorbance
0.18
0.16
0.14
0.12
0.10
0.08
0.06
0.04
0.02
0.00
3500
3000
2500
2000
1500
1000
500
Indigo-POPD (theoretical)
Absorbance
10000
8000
6000
4000
2000
0
-2000
3500
3000
2500
2000
1500
1000
500
Wavenumber (cm-1)

(d)

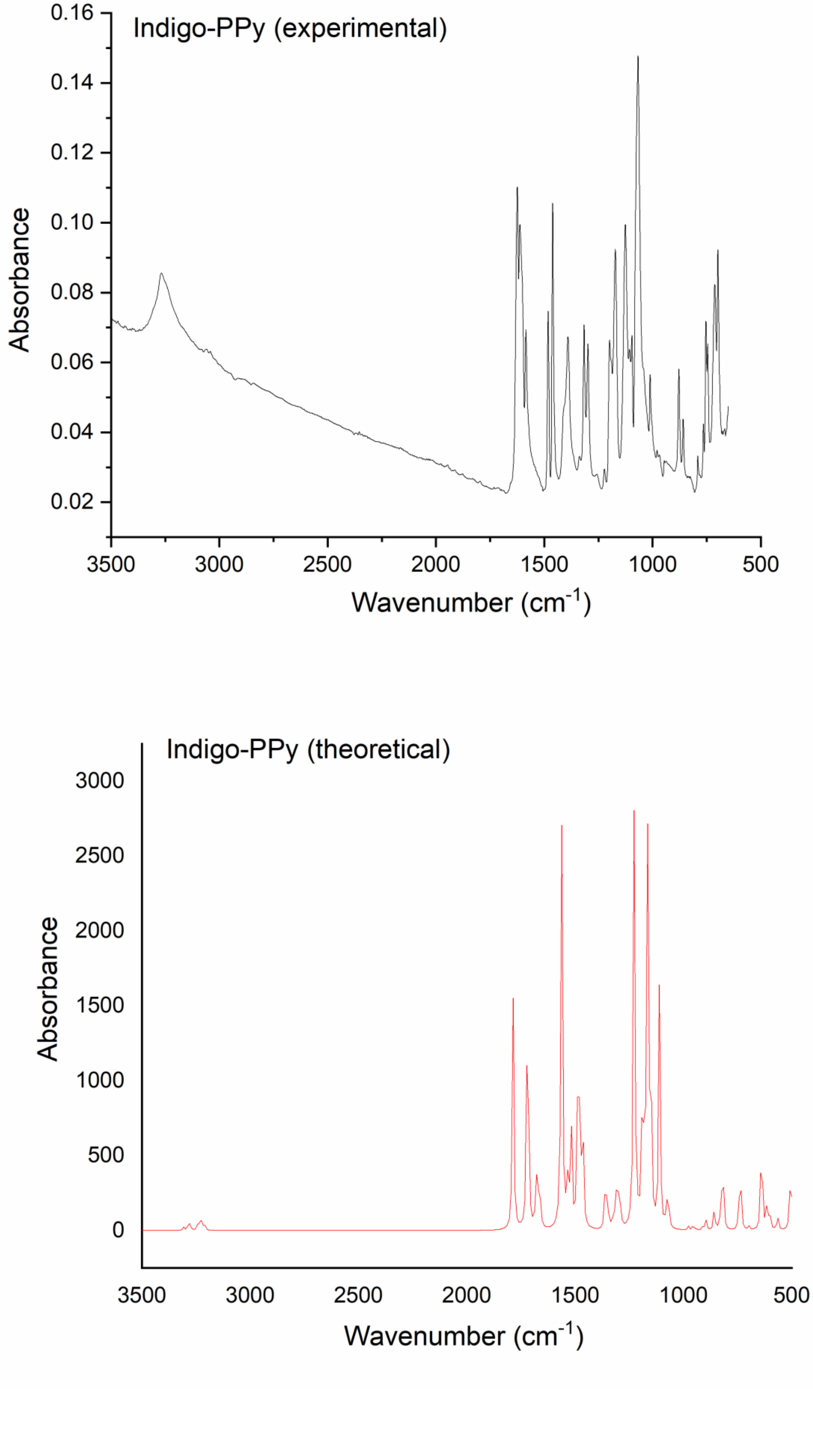
Indigo-PPy (experimental)
Absorbance
0.16
0.14
0.12
0.10
0.08
0.06
0.04
0.02
3500
3000
2500
2000
1500
1000
500
Wavenumber (cm-1)
Indigo-PPy (theoretical)
Absorbance
3000
2500
2000
1500
1000
500
0
3500
3000
2500
2000
1500
1000
500
Wavenumber (cm-1)

(e)

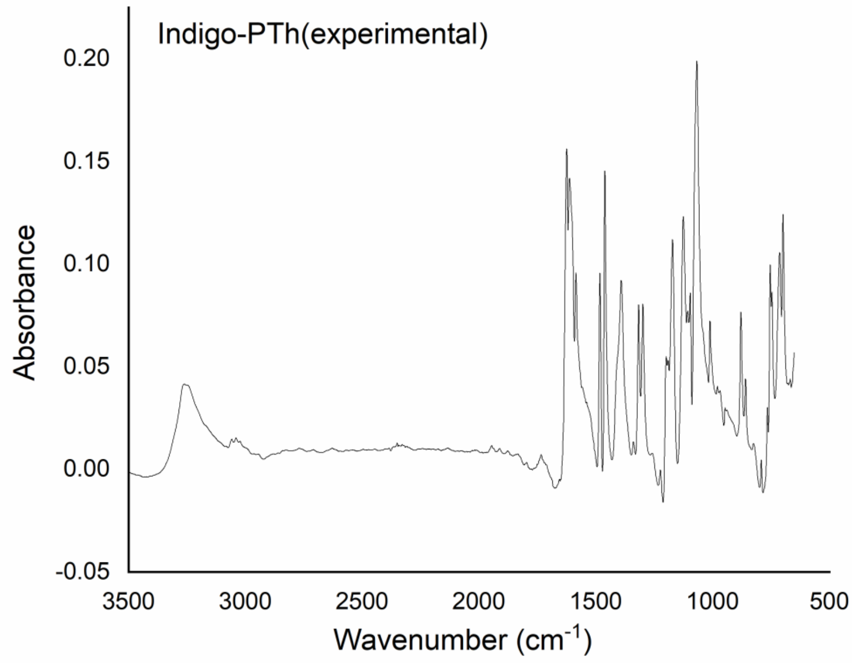

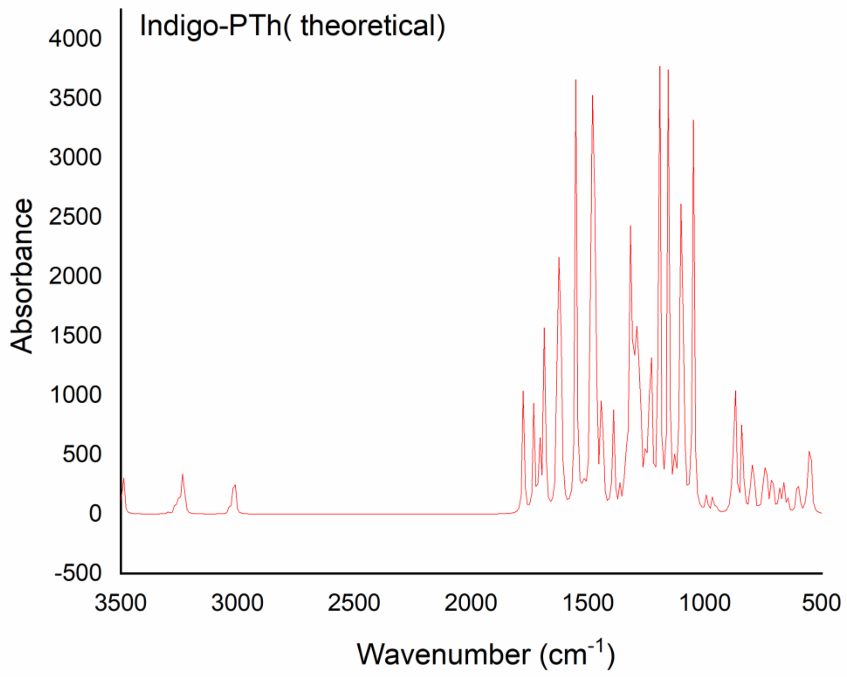

Figure S3. experimental and theoretical IR spectra of (a) Indigo-PANI, (b) Indigo-PNA, (c) Indigo-OPD, (d) Indigo-PPy, (e) Indigo-PTh

Table S1. IR spectra data of Indigo-PANI, Indigo-PNA, Indigo-POPD, Indigo-PPy and Indigo-PTh.

Polymer	Functional Group	Peak Position (cm^{-1})	
		Experimental	Theoretical
**Indigo-PANI**	N-H Stretching	3257,3035	3260,3030
	C=O Stretching	1620	1624
	C=C Stretching	1613	1610
	C=C Stretching (Quinoid)	1585 ,1483,1456	1580,1480,1455
	C=C Stretching (Benzenoid)	1390 ,1336, 1313,1298	1388,1346, 1310,1290
	C-N Stretching	1256	1250
	C-H bending	1198, 1170, 1124, 1104, 1092, 1065 1010	1190,1168,1125 1110,1088,1060 1010
	Aromatic ring stretching	934 , 876 , 857, 820, 789, 764, 753, 744 , 711, 698, 670,656	937,876,855,820,788, 760,750,745,710,695, 666,655
Indigo-PNA	N-H Stretching	3249,3056	3245,3050
	C=O Stretching	1623	1620
	C=C Stretching	1610	1615
	C=C Stretching (Quinoid)	1586	1580,1575,1552
	C=C Stretching (Benzenoid)	1391,1334,1315,1296	1390,1330,1310,1290
	C-N Stretching	1222	1220
	C-H bending	1186, 1167, 1123, 1105, 1092 1064, 1006	1184,1165,1120 1102,1087 1066,1010
	Substituted aromatic ring	975,960,946, 873, 858, 825, 791, 761, 751, 744, 710, 695, 662	970,955,945,878 850,820,790,760, 755,748,710,690, 660
Indigo-POPD	N-H Stretching	3255, 3030	3257,3030
	C=O Stretching	1622	1620
	C=C Alkene Stretching	1609	1600
	C=C Stretching (Quinoid)	1586,1482,1457	1580,1480,1450
	C=C Stretching (Benzenoid)	1389, 1337, 1317,1297	1380.1330,1310 1290
	C-N Stretching	1249, 1221	1250,1220
	C-H bending	1198, 1185, 1170, 1094, 1064, 1009	1196,1183,1168 1090,1066,1010
	C=C bending Alkene Disubstituted (trans)	980,965	978,960
	substituted aromatic ring	940, 905, 877, 858, 822, 788, 763, 750, 741, 710, 696, 673, 664	938.908,879,860 830,790,760,755 740,703,695,670 640
Indigo-PPy	N-H Stretching	3268	3259
	C=O Stretching	1625	1620
	C=C Alkene Stretching	1610	1608
	C=C Stretching (Quinoid)	1585, 1483,1462	1580,1482,1460

	C=C Stretching (Benzenoid)	1390,1338, 1314, 1298	1388,1335,1310 1290
	C-N Stretching	1254, 1220	1251,1219
	C-H bending	1198, 1172, 1123, 1104, 1095, 1066, 1010	1190,1170,1121 1110,1086,1065 1009
	C=C bending Alkene Disubstituted (trans)	978, 965	976,963
	substituted aromatic ring	945, 876, 857, 788, 764, 753, 743, 712, 698, 675, 667	940,875,850,780, 760,750,744,711 699,676,665
Indigo-PTh	N-H Stretching	3254	3250
	C=O stretching	1731, 1623	1730,1622
	C=C Alkene Stretching	1611	1610
	C=C Stretching (Quinoid)	1582, 1482, 1457,	1580,1480,1450
	C=C Stretching (Benzenoid)	1391, 1335, 1314,1296,	1390,1330,1310,1290
	C-N Stretching	1255, 1223	1250,1221
	C-H bending	1197, 1190, 1171, 1124, 1105, 1093, 1066, 1009	1195,1190,1169,1120 1102,1088,1064,1010
	C=C bending Alkene Disubstituted (trans)	978,966	977,960
	substituted aromatic ring	945, 876, 856, 822, 790, 765, 752, 743, 712, 697, 665	940,875,850,820 788,760,750,742 710,695,660
